# Effect of melatonin supplementation on cardiometabolic risk factors, oxidative stress and hormonal profile in PCOS patients: a systematic review and meta-analysis of randomized clinical trials

**DOI:** 10.1186/s13048-024-01450-z

**Published:** 2024-07-04

**Authors:** Somayeh Ziaei, Motahareh Hasani, Mahsa Malekahmadi, Elnaz Daneshzad, Katayoun Kadkhodazadeh, Javad Heshmati

**Affiliations:** 1 ICU Department, Emam Reza Hospital, Kermanshah University of Medical Sciences, Kermanshah, Iran; 2 Department of Nutritional Sciences, School of Health, Golestan University of Medical Sciences, Gorgan, Iran; 3 Imam Khomeini Hospital Complex, Tehran University of Medicinal Sciences, Tehran, Iran; 4 Non-Communicable Diseases Research Center, Alborz University of Medical Sciences, Karaj, Iran; 5 Department of Cellular and Molecular Nutrition, School of Nutritional Sciences and Dietetics, Tehran University of Medical Sciences, Tehran, Iran; 6 University of Ottawa Heart Institute, Ottawa, Canada

**Keywords:** Melatonin, PCOS, Cardiometabolic risk factors, Hormones, Pregnancy

## Abstract

**Background:**

To investigate whether melatonin supplementation can enhance cardiometabolic risk factors, reduce oxidative stress, and improve hormonal and pregnancy-related factors in patients with PCOS.

**Methods:**

We conducted a systematic search of PubMed/Medline, Scopus, and the Cochrane Library for articles published in English from inception to March 2023. We included randomized controlled trials (RCTs) on the use of melatonin for patients with polycystic ovary syndrome (PCOS). We performed a meta-analysis using a random-effects model and calculated the standardized mean differences (SMDs) and 95% confidence intervals (CIs).

**Results:**

Six studies met the inclusion criteria. The result of meta-analysis indicated that melatonin intake significantly increase TAC levels (SMD: 0.87, 95% CI: 0.46, 1.28, I^2^ = 00.00%) and has no effect on FBS, insulin, HOMA-IR, TC, TG, HDL, LDL, MDA, hs-CRP, mFG, SHBG, total testosterone, and pregnancy rate in patients with PCOS compare to controls. The included trials did not report any adverse events.

**Conclusion:**

Melatonin is a potential antioxidant that may prevent damage from oxidative stress in patients with PCOS. However, the clear effect of melatonin supplementation on cardiometabolic risk factors, hormonal outcomes, and pregnancy-related outcomes needs to be evaluated further in large populations and long-term RCTs.

**Supplementary Information:**

The online version contains supplementary material available at 10.1186/s13048-024-01450-z.

## Highlights

Melatonin supplementation increases total antioxidant capacity (TAC) levels in PCOS patients, suggesting its potential as an antioxidant therapy

Meta-analysis reveals no significant impact of melatonin on cardiometabolic risk factors, hormonal levels, or pregnancy rate in PCOS patients compared to controls

Melatonin intake shows no adverse events in PCOS patients, indicating its safety for supplementation

Further large-scale and long-term randomized controlled trials are needed to determine the full effects of melatonin supplementation in PCOS patients

**Supplementary Information:**

The online version contains supplementary material available at 10.1186/s13048-024-01450-z.

## Introduction

Polycystic ovarian syndrome (PCOS), a common endocrine disorder affecting a significant number of women before menopause, is mainly recognized by reproductive irregularities, increased androgen levels, and disrupted ovulation, impacting around 10% of premenopausal females [1]. Although the precise pathophysiology of PCOS is still not fully understood, evidences point to hyperandrogenism as having a major impact on this illness [2, 3]. The aberrant inflammatory reaction of ovarian theca cells to free oxygen radicals may lead to hyperandrogenism [4]. Furthermore, studies has indicated that PCOS is characterized by an ongoing condition of persistent mild inflammation and oxidative stress, which is strongly associated with further clinical and metabolic irregularities [5]. It is currently thought that PCOS is a condition characterized by oxidative stress and lower levels of antioxidants [6]. The presence of common insulin resistance and dyslipidemia can result in an overabundance of reactive oxygen species (ROS) due to endoplasmic reticulum stress and lipid peroxidation [7]. The activation of redox-sensitive transcription factors can be affected by oxidative stress, while the presence of lipid peroxidation might exacerbate the effects of PCOS and interfere with the regulation of glucose and lipid metabolism [8]. The development of PCOS in individuals experiencing oxidative stress might be associated with a lack of antioxidants, such as melatonin (N-acetyl-5-methoxytryptamine) [9].

The pineal gland is responsible for the primary release of melatonin [10]. Controlling sleep patterns and adjusting circadian rhythms are primary functions of the periodic release of melatonin into the bloodstream [11], and participation in the immunological response of the body [12]. Melatonin is a potent radical scavenger and an endogenous antioxidant [13]. dditionally, it plays a significant role in anti-inflammatory activities [14]. According to current studies, it appears that melatonin plays a role in different ovarian activities such as the growth of follicles, the functioning of the corpus luteum, the production of steroids, and the maturation of oocytes [15]. Melatonin appears to protect the oocyte from damage caused by ROS [16].

Moreover, the lack of melatonin appears to contribute to the underlying mechanisms of PCOS. Additionally, melatonin has demonstrated its ability to serve as a reliable indicator of oocyte quality, as a sufficient level of melatonin is positively linked to the appropriate quality of oocytes [17]. A randomized controlled trial revealed that the combined usage of melatonin and myo-inositol leads to a synergistic improvement in the quality of embryos and oocytes in women diagnosed with PCOS, who are undergoing in vitro fertilization (IVF), when compared to using myo-inositol alone [18]. Furthermore, it was found that the administration of melatonin over a duration of 12 weeks led to decreased concentrations of C-reactive protein (hs-CRP) and plasma malondialdehyde (MDA) among women diagnosed with PCOS. Simultaneously, melatonin supplementation increased total antioxidant capacity (TAC) levels and glutathione (GSH) levels. Additionally, melatonin was observed to reduce the expression of interleukin-1 (IL-1) and tumor necrosis factor alpha (TNF-α) genes [19]. However, there is no systematic review and meta-analysis that summarizes the effect of melatonin in patients with PCOS. Therefore, a systematic review was conducted to evaluate the effects of melatonin on cardiometabolic risk factors, oxidative stress, inflammatory factors, and hormonal profiles in women diagnosed with PCOS.

## Methods

### Search strategy

This systematic review and meta-analysis was undertaken based on the Preferred Reporting Items for Systematic Reviews and Meta-Analyses (PRISMA) guidelines and Cochrane handbook for systematic reviews of interventions [20]. Two authors independently searched various electronic databases, including MEDLINE, Scopus, and the Cochrane Library, from inception to April 2023. The following search terms were used in this systematic review: “Melatonin” OR “Pineal hormone” and terms related to PCOS (including MeSH search using “Polycystic Ovary Syndrome”, “PCOS” and terms related to study design (such as “Randomized controlled trial”, “Controlled Clinical Trial”, “Randomized”, “Randomly”, “Placebo”, “Trial”). The searches conducted were restricted to humans only, but not restricted based on the language. Additionally, the references of original published articles and reviews, as well as input from experts, were also explored during the search process. The complete search strategy, key terms, and syntaxes for searching each individual database are presented in Supplementary File 1.

### Inclusion and exclusion criteria inclusion criteria

The studies met the inclusion criteria if they had the following characteristics: (I) The research was conducted as a crossover or parallel randomized controlled trial (RCT). (II) The participants in the research were females with polycystic ovary syndrome (PCOS) of any age. (III) The intervention group was given melatonin alone or in combination with other treatments, while the control group received no treatment or only other treatments. (IV) The primary outcome of the article consists of at least one of the following factors: anthropometric indices such as weight, body mass index (BMI), waist circumference (WC), hip circumference (HC), and glycemic parameters such as fasting blood sugar (FBS), insulin, homeostatic model assessment of insulin resistance (HOMA-IR), and lipid profile parameters including total cholesterol (TC), triglycerides (TG), high-density lipoprotein (HDL), low-density lipoprotein (LDL), as well as oxidative stress and inflammatory indicators such as total antioxidant capacity (TAC), malondialdehyde (MDA), and high-sensitivity C-reactive protein (hs-CRP), and finally hormonal and pregnancy-related factors such as total testosterone, sex hormone-binding globulin (SHBG), Modified Ferriman–Gallwey Score (mFG), endometrial thickness, and pregnancy rate. The process involved excluding literature reviews, observational studies, case reports, and molecular and animal studies. Following this, duplicate studies were removed, and two reviewers individually assessed the remaining studies for inclusion based on their titles, abstracts, and full texts if necessary.

### Data extraction and quality appraisal

Two reviewers extracted data separately, including study details and primary results. In case of any inconsistencies, they resolved them by discussing with a third author. Additionally, information such as the country of origin, dose and duration of melatonin intake, as well as patient-related details like age and the number of patients, were also gathered. The collected data included the means, the standard deviation of those means, and the number of participants in each group. If precise data was not provided, such as with graphs or bar charts, we requested unpublished data from the author. If we could not obtain this information, we used a digital ruler to estimate the data from the graphs or charts. The included studies were appraised and graded independently by two reviewers according to the Cochrane risk of bias evaluation tool [21].

### Meta-analytic methods

We performed meta-analyses utilizing Stata software version 17.0 (Stata Corp, College Station, TX, USA). We assessed the influence of melatonin intervention using the standardized mean difference (SMD) of the variables of interest. The results for continuous variables were presented as effect sizes along with confidence intervals (CIs). A random-effects model was used to calculate the effect sizes of the variables of interest and a meta-analysis was conducted. We considered data from intention to-treat analyses. The heterogeneity of the study was investigated using the Chi-square test of homogeneity (*p* < 0.05) in addition to the I^2^ statistic. A level of I^2^ ≥ 50% was considered indicative of a high level of heterogeneity.

## Results

### Study selection

**Fig. 1:**
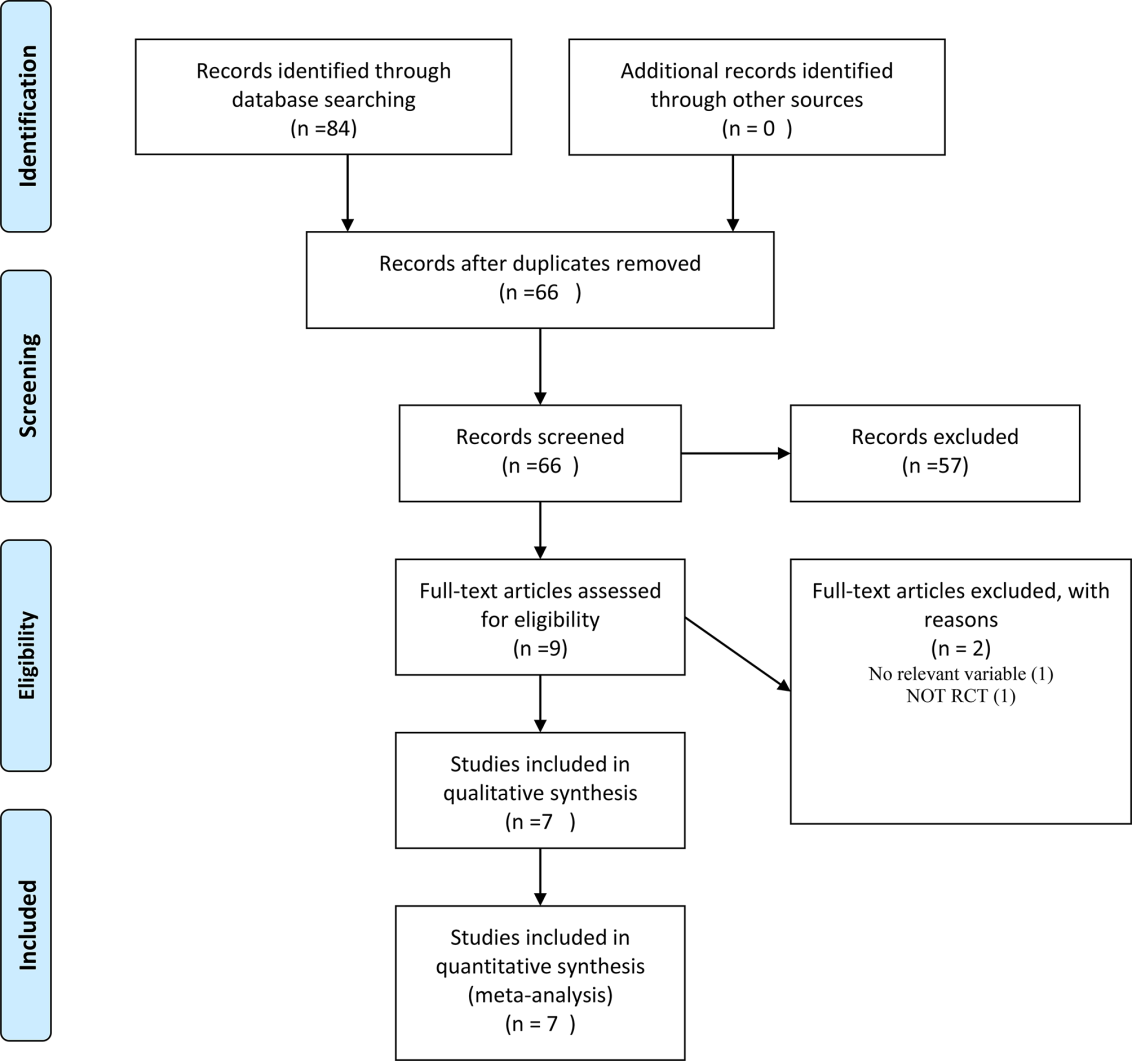
PRISMA flow diagram of study selection

The primary search identified 84 articles from searched databases. After duplicate removal, there were 66 records for screening of title and abstract. Nine full texts were obtained for final screening, and out of those nine, three were excluded because they did not evaluate relevant variables (*n* = 2) and were not RCTs (*n* = 1). Finally, six trials met the inclusion criteria for this systematic review and meta-analysis [5, 18, 19, 22–24]. The PRISMA flow diagram of the studies selection is presented in Fig. 1.

### Study characteristics

**Fig. 2:**
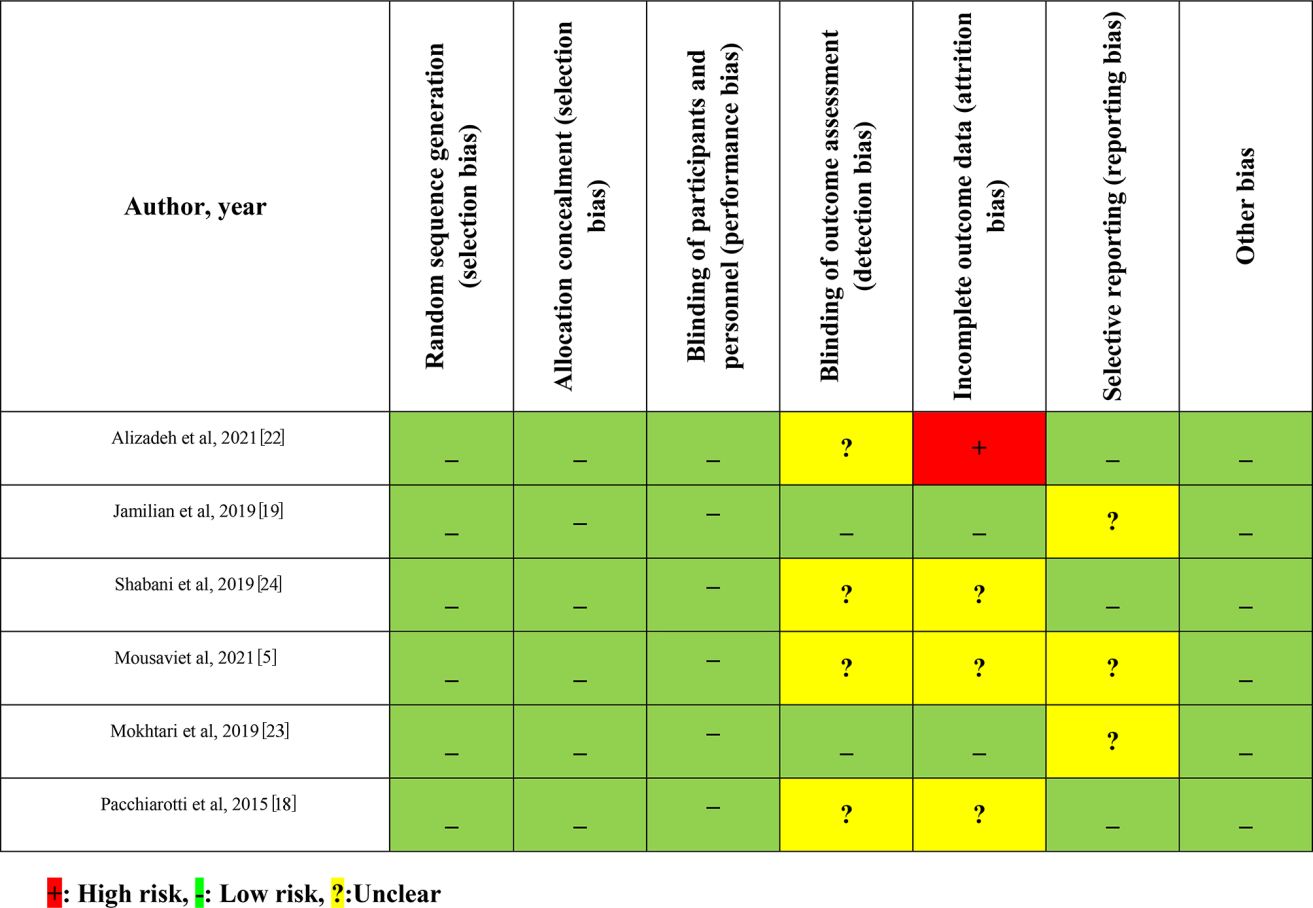
Risk of bias assessment of included trials [5, 18, 19, 22–24]

The included studies characteristics and findings are shown in Table 1. All six studies, which were included in this systematic review, were double-blind RCTs published between 2015 and 2021. These studies were conducted in Iran and Italy. The trials that were included had 1006 participants who were administered melatonin orally, with doses ranging from 3 to 10 mg/day. The age of participants varied between 25 and 31 years, and their BMI ranged from 22 to 29 kg/m^2^, while the duration of the intervention lasted between 3 and 12 weeks. All the included trials were evaluated as being of good quality, and the report of the quality appraisal for these trials is presented in Fig. 2. Included studies did not provide any information on adverse events.

**Table 1:** Main characteristics of included studies

Study (ref)	Country	Sample size	Melatonin dosage (mg/day)	Duration (week)	Age (years)		BMI (kg/m^2^)		Main outcome^§^
					Intervention Mean ± SD	Placebo Mean ± SD	Intervention Mean ± SD	Placebo Mean ± SD	
Alizadeh et al., 2021 [22]	Iran	84	6	8	25.57 ± 4.99	26.20 ± 5.72	28.40 ± 3.86	26.94 ± 3.83	↑HDL, ↔BMI, ↔WC, ↔FBS, ↔Ins, ↔HOMA-IR, ↔HOMA-B, ↔TC, ↔TG, ↔LDL, ↔Total Testosterone, ↔SHBG, ↔mFG
Jamilian et al., 2019 [19]	Iran	56	10	12	28.7 ± 2.1	28.3 ± 2.3	29.1 ± 4.6	29.2 ± 3.5	↓mFG, ↓Total Testosterone, ↓hs-CRP, ↓MDA, ↑TAC, ↑GSH
Shabani et al., 2019 [24]	Iran	58	10	12	26.5 ± 3.5	26.0 ± 3.3	27.1 ± 4.6	27.8 ± 4.7	↓Ins, ↓HOMA-IR, ↓TC, ↓LDL,↑QUICKI, ↔FBS, ↔TG, ↔VLDL
Mousaviet al, 2021 [5]	Iran	84	6	8	25.57 ± 4.99	26.20 ± 5.70	28.40 ± 3.86	26.94 ± 3.83	↑TAC, ↔mFG, ↔hs-CRP, ↔MDA
Mokhtari et al., 2019 [23]	Iran	198	3	3	28.4 ± 5.5	29.3 ± 5.6	27.6 ± 4.0	28.1 ± 3.7	↑Chemical pregnancy, ↑Endometrial thickness
Pacchiarotti et al., 2015 [18]	Italy	526	3	3	31.2 ± 2.1	31.5 ± 2.8	22.8 ± 1.3	23.1 ± 1.7	↑oocyte and embryo quality,

symbol is a sign of decreasing variables in the intervention group

↑This symbol is a sign of increasing variables in the intervention group

↔This sign indicates that there is no difference between the two groups. NR: not reported

§INS: Insulin, HOMA-IR: homeostatic model assessment-insulin resistance, HOMA-B: homeostatic model assessment-Beta cell function, QUICKI: quantitative insulin sensitivity check index, TG: triglycerides, VLDL: Very Low Density Lipoproteins, HDL: High Density Lipoproteins, LDL: Low Density Lipoproteins, TC: Total Cholesterol, FBS: Fasting blood Sugar, hs-CRP: high-sensitivity C-reactive protein, MDA: Malondialdehyde, TAC: Total Antioxidant Capacity, GSH: glutathione, NO: nitric oxide, SHBG: sex hormone-binding globulin, FSH: Follicle-Stimulating Hormone, LH: Luteinizing Hormone

### Meta-analysis

**Fig. 3:**
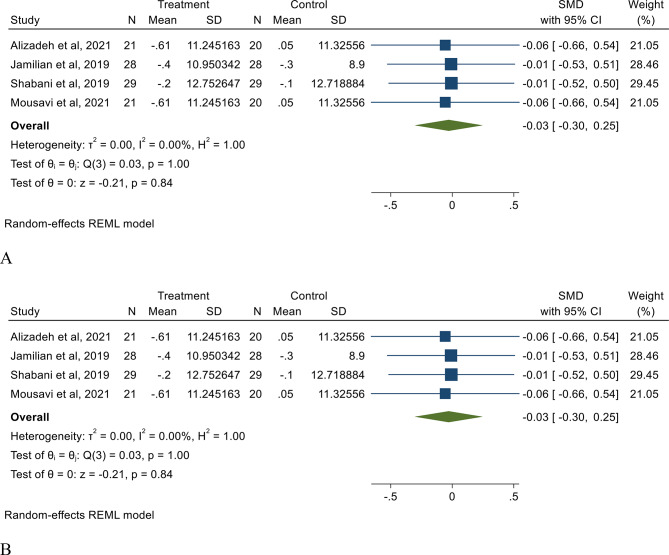
The effect of melatonin intake on weight (**A**), and BMI (**B**) in PCOS patients compare to placebo

The results of random-effect meta-analysis indicated that melatonin intake has no effect on weight (SMD: −0.03, 95% CI: −0.30, 0.25, I^2^ = 00.00%) and BMI (SMD: −0.03, 95% CI: −0.30, 0.25, I^2^ = 00.00%) in patients with PCOS (Fig. 3.). The results of subgroup and sensitivity analysis showed no significant difference. The result of meta-analysis also indicated that melatonin intake has no effect on FBS, insulin, HOMA-IR, TC, TG, HDL, LDL, MDA, and hs-CRP. Our meta-analysis indicated that melatonin intake significantly increase TAC levels (SMD: 0.87, 95% CI: 0.46, 1.28, I^2^ = 00.00%) compare to placebo (Table 2). Additionally, while the impact of melatonin intake on mFG, SHBG, total testosterone, endometrial thickness, and pregnancy rate was not statistically significant, a marginal decrease in total testosterone and a slight increase in endometrial thickness were detected (Table 2). We investigate the source of heterogeneity in our meta-analysis by performing a sensitivity analysis. The results of the sensitivity analysis are presented in Supplementary File 2.

**Table 2:** Meta-analysis of effect of melatonin in PCOS patients

	Variable	effect size SMD	95% CI	I^2^ (%)	*P* for heterogeneity
Glycemic parameters	FBS	-0.15	-0.54, 0.24	00.00	0.450
	Insulin	-0.35	-0.99, 0.28	60.99	0.280
	HOMA-IR	-0.33	-0.93, 0.27	56.29	0.280
Lipid Profile	TC	-0.04	-0.60, 0.52	50.66	0.890
	TG	-0.01	-0.38, 0.39	00.00	0.980
	HDL	0.06	-0.33, 0.45	00.00	0.770
	LDL	-0.07	-0.53, 0.39	27.45	0.760
Oxidative Stress and Inflammation	TAC	**0.87**	0.46, 1.28*	00.00	**0.000**
	MDA	0.08	-0.91, 1.08	83.62	0.870
	hs-CRP	-0.22	-0.61, 0.17	00.00	0.270
Hormonal and pregnancy outcomes	mFG	0.15	-0.54, 0.24	00.00	0.460
	SHGB	0.10	-0.29, 0.49	00.00	0.620
	Total Testosterone	-0.25	0.65, 0.14	00.00	0.210
	Endometrial thickness	0.23	0.53, 0.99	95.01	0.550
		**RR**	**95% CI**	**I** ^**2**^ **(%)**	**P for heterogeneity**
	Pregnancy Rate	0.30	-0.16, 0.77	56.60	0.200

*SMD: Standard mean difference, CI: confidence interval, FBS: Fasting Blood Sugar, HOMA-IR: homeostatic model assessment-insulin resistance, TC: Total Cholesterol, TG: triglycerides, HDL: High Density Lipoproteins, LDL: Low Density Lipoproteins, hs-CRP: high-sensitivity C-reactive protein, MDA: Malondialdehyde, TAC: Total Antioxidant Capacity, SHBG: sex hormone-binding globulin, mFG: Modified Ferriman–Gallwey Score*

*statistically significant

## Discussion

Our current systematic review and meta-analysis entails compiling data from six RCTs that investigate the effects of melatonin supplementation on cardiometabolic risk factors, including anthropometric indices, glycemic parameters, lipid profile, oxidative stress, and inflammatory factors, as well as hormonal and pregnancy outcomes, in patients with PCOS. The primary outcome of the present research indicated that melatonin supplementation led to a significant rise in TAC levels in comparison to the placebo group. Nevertheless, there was a slight overall decrease in hs-CRP and total testosterone levels. To the best of our knowledge, this is the first comprehensive evaluation and meta-analysis that examines the effects of melatonin on individuals diagnosed with PCOS. However, there is evidence in the literature from systematic reviews that shows melatonin concentration is reduced in patients with PCOS [25]. It has also been demonstrated that melatonin supplementation can be effective in increasing pregnancy rates in infertile women who undergo ART techniques [26].

The results of the current systematic review and meta-analysis indicate that melatonin supplementation has no effect on glycemic parameters, including FBS, insulin, and HOMA-IR, in patients with PCOS. These findings are not in accordance with previous systematic reviews and meta-analyses regarding the effect of melatonin on glycemic control and diabetes. Previous studies have shown that melatonin can decrease FBS levels [27, 28]. The limited number of trials included in the current systematic review and the differing pathophysiology of PCOS and diabetes may have prevented us from finding a significant effect of melatonin on FBS. However, previous systematic reviews have also supported the notion that melatonin intake does not have an effect on HOMA-IR and insulin levels [27, 28]. The results of the current systematic review and meta-analysis indicate that melatonin intake has no effect on lipid profiles, including TC, TG, HDL, and LDL, in patients with PCOS. Previous systematic review and meta-analyses on the effects of melatonin on lipid profiles have indicated that melatonin can decrease TC and TG levels, but has no effect on HDL and LDL [29]. The results of other systematic reviews in the field indicate that intake of melatonin can decrease levels of LDL and TG, while having no effect on TC and HDL levels [30]. It appears that the controversial outcomes of prior studies and the current systematic review highlight the necessity to assess the impact of melatonin on lipid profile in patients with PCOS through larger and longer-term RCTs.

The results of our systematic review indicate that melatonin supplementation significantly increases TAC levels and has no effect on MDA and hs-CRP in patients with PCOS. These findings are consistent with previous reviews regarding the effect of melatonin on oxidative stress parameters [31]. Based on the available data, it appears that melatonin possesses antioxidant protective properties through both its direct free radical scavenging ability and its indirect antioxidant activity [32, 33]. It has been demonstrated that Melatonin effectively interacts with different reactive oxygen and reactive nitrogen species [34]. Moreover, it upregulates antioxidant enzymes while downregulating pro-oxidant enzymes [35]. It has also been demonstrated that melatonin decreases the generation of ROS through its effect on TNF-α gene expression [36, 37]. TNF-α can increase the expression of NOX by activating NF-κB signaling, thereby inducing the generation of ROS [38, 39]. The production and removal of ROS are primarily influenced by the equilibrium between oxidase and antioxidase. Major sources of ROS include NADPH oxidative enzymes, such as NOX1 and NOX2 [40, 41]. The expression of NADPH oxidative enzymes is upregulated by the presence of inflammatory cytokines, leading to increased proliferation of ROS [42]. The presence of TNF-α led to a significant elevation in the expression of both NOX1 and NOX2 [43]. Recent findings have revealed that melatonin can significantly reduce the production of ROS, which was previously increased due to TNF-α induction [44]. It has also been demonstrated that melatonin can lead to the downregulation of NOX1 and NOX2 expression, as well as the upregulation of primary antioxidant enzymes such as SOD1 and CAT expression [45, 46]. It has also been reported that melatonin can decrease ROS production by suppressing the expression of the NF-kB gene [47].

The results of the current meta-analysis indicate no effect of melatonin supplementation on hormonal and pregnancy outcomes in patients with PCOS. However, there was a marginal trend towards decreasing androgens and increasing endometrial thickness after melatonin intake in women with PCOS. Melatonin is an important hormone that plays a crucial role in regulating the development of follicles in the ovaries [48]. The presence of receptors MT1 and MT2 in the ovarian follicle has been shown by multiple authors, which supports the idea that melatonin has a role in the functioning of the ovaries [49]. It has been demonstrated that the administration of melatonin can increase the synthesis of insulin-like growth factor I (IGF-I), a significant growth factor in granulosa cells that promotes follicular development [50]. Recent research has shown that melatonin at a concentration of 0.1 mM can affect the expression of IGF-I receptors and may induce the development of primary and antral follicles [51]. Recent findings have discovered that melatonin is present in the follicular fluid of preovulatory follicles in humans, and its levels in this fluid are higher than those in serum [52]. The elevated concentrations of melatonin in follicular fluid are believed to be crucial for the growth and appropriate maturation of ovarian follicles, whereas low levels may result in anovulation and inferior oocyte quality in women with PCOS [53].

The role of melatonin is crucial as it serves as an antioxidant, antiapoptotic, and anti-inflammatory factor [31, 54]. It has the potential to protect the oocyte and surrounding cells from oxidative stress damage, thereby preventing follicular atresia [17, 55]. Research findings suggest that oxidative stress can harm granulosa cells, leading to an increased rate of apoptosis and damaging oocyte DNA [56]. Studies also indicate that melatonin treatment resulted in a higher number of antral and primary follicles compared to the control group [57, 58]. Furthermore, administering melatonin to animals before induction of permanent estrus state showed a higher number of ovarian follicles [59].

This study, which is the initial extensive meta-analysis, investigates the impact of melatonin supplementation on factors related to cardiometabolic health, oxidative stress, and hormones in individuals diagnosed with PCOS. Nevertheless, there were certain constraints associated with this research. Primarily, a majority of the trials examined in this meta-analysis consisted of a limited number of participants, and the overall count of studies incorporated was relatively small. Theoretically, this limitation could result in uncertain evaluations of treatment outcomes. Secondly, the included trials were limited to only two specific countries of origin, which limits the generalizability of our findings. Finally, wide heterogeneity was detected between the included trials, and due to the limited number of included studies, we are unable to perform subgroup analysis to deal with this high heterogeneity.

## Conclusion

The current meta-analysis demonstrated that the addition of melatonin as a supplement led to a substantial elevation in TAC levels among individuals diagnosed with PCOS. However, it had no significant effects on other cardiometabolic and hormonal factors, as well as pregnancy-related factors in these patients. It seems that large population and long-duration RCTs are still needed to draw a clear conclusion about the effect of melatonin supplementation in patients with PCOS.

### Electronic supplementary material

Below is the link to the electronic supplementary material.

Supplementary Material 1

Supplementary Material 2

## Data Availability

Not applicable.

## Abbreviations

SMD: Standard Mean Difference
CI: Confidence Interval
FBS: Fasting Blood Sugar
HOMA-IR: Homeostatic Model Assessment-insulin Resistance
TC: Total Cholesterol
TG: Triglycerides
HDL: High Density Lipoproteins
LDL: Low Density Lipoproteins
hs-CRP: High-sensitivity C-reactive Protein
MDA: Malondialdehyde
TAC: Total Antioxidant Capacity
SHBG: Sex Hormone-binding Globulin
mFG: Modified Ferriman–Gallwey Score
