# Supplementary material for: Effect of melatonin supplementation on cardiometabolic risk factors, oxidative stress and hormonal profile in PCOS patients: a systematic review and meta-analysis of randomized clinical trials
Source: J Ovarian Res. 2024 Jul 4;17:138. doi: 10.1186/s13048-024-01450-z (PMC11225253; doi:10.1186/s13048-024-01450-z)
Supplement: Supplementary file 1 — Supplementary Material 1 [file 13048_2024_1450_MOESM1_ESM.doc]

**Supplementary File 1**

**A systematic review and meta-analysis of the Melatonin effects on PCOS patients**

| **Groups** | **Descriptors** |
| --- | --- |
| Outcome | Polycystic Ovary Syndrome OR Ovary Syndrome, Polycystic OR Syndrome, Polycystic Ovary OR Polycystic ovary disease OR Stein-Leventhal Syndrome OR Stein Leventhal Syndrome OR Syndrome, Stein-Leventhal OR Sclerocystic Ovarian Degeneration OR Ovarian Degeneration, Sclerocystic OR Sclerocystic Ovary Syndrome OR Polycystic Ovarian Syndrome OR Ovarian Syndrome, Polycystic OR Polycystic Ovary Syndrome 1 OR Sclerocystic Ovaries OR Ovary, Sclerocystic OR Sclerocystic Ovary OR hyperandrogenism OR Hypertrichosis OR Hirsutism OR “PCOS” OR “PCO” |
| Exposure | Melatonin OR Pineal hormone |
| Setting | Randomized controlled trial OR controlled clinical trial OR randomized controlled trials OR random allocation OR double blind method OR single blind method OR clinical trial OR clinical trials OR placebos OR placebo OR random |

**PUBMED**

**Number of localized studies:** 12

**Limits:** no limit

**Number of studies after applying limits:** 12

|  | **Descriptors** | Number of studies reached |
| --- | --- | --- |
| **#1** | ((((((((((((((((((("polycystic ovary syndrome"[MeSH Terms] OR ("polycystic"[All Fields] AND "ovary"[All Fields] AND "syndrome"[All Fields]) OR "polycystic ovary syndrome"[All Fields]) OR ("polycystic ovary syndrome"[MeSH Terms] OR ("polycystic"[All Fields] AND "ovary"[All Fields] AND "syndrome"[All Fields]) OR "polycystic ovary syndrome"[All Fields] OR ("ovary"[All Fields] AND "syndrome"[All Fields] AND "polycystic"[All Fields]))) OR ("polycystic ovary syndrome"[MeSH Terms] OR ("polycystic"[All Fields] AND "ovary"[All Fields] AND "syndrome"[All Fields]) OR "polycystic ovary syndrome"[All Fields] OR ("polycystic"[All Fields] AND "ovary"[All Fields] AND "disease"[All Fields]) OR "polycystic ovary disease"[All Fields])) OR ("polycystic ovary syndrome"[MeSH Terms] OR ("polycystic"[All Fields] AND "ovary"[All Fields] AND "syndrome"[All Fields]) OR "polycystic ovary syndrome"[All Fields] OR ("stein"[All Fields] AND "leventhal"[All Fields] AND "syndrome"[All Fields]) OR "stein leventhal syndrome"[All Fields])) OR ("polycystic ovary syndrome"[MeSH Terms] OR ("polycystic"[All Fields] AND "ovary"[All Fields] AND "syndrome"[All Fields]) OR "polycystic ovary syndrome"[All Fields] OR ("stein"[All Fields] AND "leventhal"[All Fields] AND "syndrome"[All Fields]) OR "stein leventhal syndrome"[All Fields])) OR ("polycystic ovary syndrome"[MeSH Terms] OR ("polycystic"[All Fields] AND "ovary"[All Fields] AND "syndrome"[All Fields]) OR "polycystic ovary syndrome"[All Fields] OR ("syndrome"[All Fields] AND "stein"[All Fields] AND "leventhal"[All Fields]))) OR ("polycystic ovary syndrome"[MeSH Terms] OR ("polycystic"[All Fields] AND "ovary"[All Fields] AND "syndrome"[All Fields]) OR "polycystic ovary syndrome"[All Fields] OR ("sclerocystic"[All Fields] AND "ovarian"[All Fields] AND "degeneration"[All Fields]) OR "sclerocystic ovarian degeneration"[All Fields])) OR ("polycystic ovary syndrome"[MeSH Terms] OR ("polycystic"[All Fields] AND "ovary"[All Fields] AND "syndrome"[All Fields]) OR "polycystic ovary syndrome"[All Fields] OR ("ovarian"[All Fields] AND "degeneration"[All Fields] AND "sclerocystic"[All Fields]))) OR ("polycystic ovary syndrome"[MeSH Terms] OR ("polycystic"[All Fields] AND "ovary"[All Fields] AND "syndrome"[All Fields]) OR "polycystic ovary syndrome"[All Fields] OR ("sclerocystic"[All Fields] AND "ovary"[All Fields] AND "syndrome"[All Fields]) OR "sclerocystic ovary syndrome"[All Fields])) OR ("polycystic ovary syndrome"[MeSH Terms] OR ("polycystic"[All Fields] AND "ovary"[All Fields] AND "syndrome"[All Fields]) OR "polycystic ovary syndrome"[All Fields] OR ("polycystic"[All Fields] AND "ovarian"[All Fields] AND "syndrome"[All Fields]) OR "polycystic ovarian syndrome"[All Fields])) OR ("polycystic ovary syndrome"[MeSH Terms] OR ("polycystic"[All Fields] AND "ovary"[All Fields] AND "syndrome"[All Fields]) OR "polycystic ovary syndrome"[All Fields] OR ("ovarian"[All Fields] AND "syndrome"[All Fields] AND "polycystic"[All Fields]))) OR ("polycystic ovary syndrome"[MeSH Terms] OR ("polycystic"[All Fields] AND "ovary"[All Fields] AND "syndrome"[All Fields]) OR "polycystic ovary syndrome"[All Fields] OR "polycystic ovary syndrome 1"[All Fields])) OR ("polycystic ovary syndrome"[MeSH Terms] OR ("polycystic"[All Fields] AND "ovary"[All Fields] AND "syndrome"[All Fields]) OR "polycystic ovary syndrome"[All Fields] OR ("sclerocystic"[All Fields] AND "ovaries"[All Fields]) OR "sclerocystic ovaries"[All Fields])) OR ("polycystic ovary syndrome"[MeSH Terms] OR ("polycystic"[All Fields] AND "ovary"[All Fields] AND "syndrome"[All Fields]) OR "polycystic ovary syndrome"[All Fields] OR ("ovary"[All Fields] AND "sclerocystic"[All Fields]))) OR ("polycystic ovary syndrome"[MeSH Terms] OR ("polycystic"[All Fields] AND "ovary"[All Fields] AND "syndrome"[All Fields]) OR "polycystic ovary syndrome"[All Fields] OR ("sclerocystic"[All Fields] AND "ovary"[All Fields]) OR "sclerocystic ovary"[All Fields])) OR ("hyperandrogenism"[MeSH Terms] OR "hyperandrogenism"[All Fields])) OR ("hypertrichosis"[MeSH Terms] OR "hypertrichosis"[All Fields])) OR ("hirsutism"[MeSH Terms] OR "hirsutism"[All Fields])) OR PCOS[All Fields]) OR PCO[All Fields] | 37888 |
| **#2** | ("Melatonin"[Mesh]) OR Pineal hormone | 41907 |
| **#3** | ((((((((("Randomized Controlled Trial"[Publication Type] OR "Controlled Clinical Trial"[Publication Type]) OR "Randomized Controlled Trials as Topic"[Mesh]) OR "Random Allocation"[Mesh]) OR "Double-Blind Method"[Mesh]) OR "Single-Blind Method"[Mesh]) OR "Clinical Trial"[Publication Type]) OR ("clinical trial"[Publication Type] OR "clinical trials as topic"[MeSH Terms] OR "clinical trials"[All Fields])) OR "Placebos"[Mesh]) OR ("placebos"[MeSH Terms] OR "placebos"[All Fields] OR "placebo"[All Fields])) OR ("random allocation"[MeSH Terms] OR ("random"[All Fields] AND "allocation"[All Fields]) OR "random allocation"[All Fields] OR "random"[All Fields]) | 1956936 |
| **#4** | **#1** AND **#2** AND #3 | 12 |

**WEB OF SCIENCE**

**Number of localized studies:** 20

**Limits:** documents types (articles)

**Number of studies after applying limits:** 15

|  | **Descriptors** | Number of studies reached |
| --- | --- | --- |
| **#1** | TS=(“Polycystic Ovary Syndrome”) OR TS=(“Ovary Syndrome, Polycystic”) OR TS=(“Syndrome, Polycystic Ovary”) OR TS=(“Polycystic ovary disease”) OR TS=(“Stein-Leventhal Syndrome”) OR TS=(“Stein Leventhal Syndrome”) AND TS=(“Syndrome, Stein-Leventhal”) OR TS=(“Sclerocystic Ovarian Degeneration”) OR TS=(“Ovarian Degeneration, Sclerocystic”) OR TS=(“Sclerocystic Ovary Syndrome”) OR TS=(“Polycystic Ovarian Syndrome”) AND TS=(“Ovarian Syndrome, Polycystic”) OR TS=(“Polycystic Ovary Syndrome 1”) OR TS=(“Sclerocystic Ovaries”) OR TS=(“Ovary, Sclerocystic”) OR TS=(“Sclerocystic Ovary”) OR TS=(“hyperandrogenism”) OR TS=(“Hypertrichosis”) OR TS=(“Hirsutism”) OR TS=(“PCOS”) OR TS=(“PCO”) | 38405 |
| **#2** | TS=(“Melatonin”) OR TS=(“Pineal hormone”) | 34828 |
| **#3** | TS=(Randomized controlled trial) OR TS=(controlled clinical trial) OR TS=(randomized controlled trials) OR TS=(random allocation) OR TS=(double blind method) OR TS=(single blind method) OR TS=(clinical trial) OR TS=(clinical trials) OR TS=(placebos) OR TS=(placebo) OR TS=(random) | 1862477 |
| **#4** | **#1** AND **#2** AND **#3** | 20 |

**SCOPUS**

**Number of localized studies:** 50

**Limits:** *document type* (article and article in press)

**Number of studies after applying limits:** 22

|  | **Descriptors** | Number of studies reached |
| --- | --- | --- |
| **#1** | ( TITLE-ABS-KEY ( hypertrichosis ) ) OR ( TITLE-ABS-KEY ( hirsutism ) ) OR ( TITLE-ABS-KEY ( pcos ) ) OR ( TITLE-ABS-KEY ( pco ) ) OR ( ( TITLE-ABS-KEY ( polycystic AND ovary AND syndrome ) ) OR ( TITLE-ABS-KEY ( ovary AND syndrome, AND polycystic ) ) OR ( TITLE-ABS-KEY ( syndrome, AND polycystic AND ovary ) ) OR ( TITLE-ABS-KEY ( polycystic AND ovary AND disease ) ) OR ( TITLE-ABS-KEY ( stein-leventhal AND syndrome ) ) OR ( TITLE-ABS-KEY ( stein AND leventhal AND syndrome ) ) ) OR ( ( TITLE-ABS-KEY ( syndrome, AND stein-leventhal ) ) OR ( TITLE-ABS-KEY ( sclerocystic AND ovarian AND degeneration ) ) OR ( TITLE-ABS-KEY ( ovarian AND degeneration, AND sclerocystic ) ) OR ( TITLE-ABS-KEY ( sclerocystic AND ovary AND syndrome ) ) OR ( TITLE-ABS-KEY ( polycystic AND ovarian AND syndrome ) ) OR ( TITLE-ABS-KEY ( ovarian AND syndrome, AND polycystic ) ) ) OR ( ( TITLE-ABS-KEY ( polycystic AND ovary AND syndrome 1 ) ) OR ( TITLE-ABS-KEY ( sclerocystic AND ovaries ) ) OR ( TITLE-ABS-KEY ( ovary, AND sclerocystic ) ) OR ( TITLE-ABS-KEY ( sclerocystic AND ovary ) ) OR ( TITLE-ABS-KEY ( hyperandrogenism ) ) OR ( TITLE-ABS-KEY ( hypertrichosis ) ) ) | 61901 |
| **#2** | ( TITLE-ABS-KEY ( melatonin ) ) OR ( TITLE-ABS-KEY ( pineal AND hormone ) ) | 48459 |
| **#3** | ( TITLE-ABS-KEY ( randomized AND controlled AND trial ) OR TITLE-ABS-KEY ( controlled AND clinical AND trial ) OR TITLE-ABS-KEY ( randomized AND controlled AND trials ) OR TITLE-ABS-KEY ( random AND allocation ) OR TITLE-ABS-KEY ( double AND blind AND method ) OR TITLE-ABS-KEY ( single AND blind AND method ) OR TITLE-ABS-KEY ( clinical AND trial ) OR TITLE-ABS-KEY ( clinical AND trials ) OR TITLE-ABS-KEY ( placebos ) OR TITLE-ABS-KEY ( placebo ) OR TITLE-ABS-KEY ( random ) ) | 3732252 |
| **#4** | **#1** AND **#2** AND **#3** | 50 |

**COCHRANE**

**Number of localized studies:** 2

**Limits:** -

**Number of studies after applying limits:** 2

|  | **Descriptors** | Number of studies reached |
| --- | --- | --- |
| **#1** | Me ("Polycystic Ovary Syndrome"):ti,ab,kw or ("Ovary Syndrome, Polycystic"):ti,ab,kw or ("Syndrome, Polycystic Ovary"):ti,ab,kw or ("Polycystic ovary disease"):ti,ab,kw or ("Stein-Leventhal Syndrome"):ti,ab,kw or ("Stein Leventhal Syndrome"):ti,ab,kw or ("Syndrome, Stein-Leventhal"):ti,ab,kw or ("Sclerocystic Ovarian Degeneration"):ti,ab,kw or ("Ovarian Degeneration, Sclerocystic"):ti,ab,kw or ("Sclerocystic Ovary Syndrome"):ti,ab,kw or ("Polycystic Ovarian Syndrome "):ti,ab,kw or ("Ovarian Syndrome, Polycystic"):ti,ab,kw or ("Polycystic Ovary Syndrome 1"):ti,ab,kw or ("Sclerocystic Ovaries"):ti,ab,kw or ("Ovary, Sclerocystic"):ti,ab,kw or ("Sclerocystic Ovary"):ti,ab,kw or ("hyperandrogenism"):ti,ab,kw or ("Hypertrichosis"):ti,ab,kw or ("Hirsutism"):ti,ab,kw or ("PCOS"):ti,ab,kw or ("PCO"):ti,ab,kw | 4781 |
| **#2** | Me ("Melatonin") or ("Pineal hormone"):ti,ab,kw | 102 |
| **#3** | **#1** AND **#2** | 2 |

Excluded Articles

|  | **Study** | **Reason** |
| --- | --- | --- |
| 1 |  |  |
| 2 |  |  |
| 3 |  |  |
