# Supplementary material for: Effect of melatonin supplementation on cardiometabolic risk factors, oxidative stress and hormonal profile in PCOS patients: a systematic review and meta-analysis of randomized clinical trials
Source: J Ovarian Res. 2024 Jul 4;17:138. doi: 10.1186/s13048-024-01450-z (PMC11225253; doi:10.1186/s13048-024-01450-z)
Supplement: Supplementary file 2 — Supplementary Material 2 [file 13048_2024_1450_MOESM2_ESM.docx]

A

B

**Supplementary File 2.** Sensitivity analysis of the effect of melatonin on Weight (A), and BMI (B) in PCOS patients compare to placebo.
